# Portable ultra-low-field MRI for progressive multifocal leukoencephalopathy: Case studies, sensitivity, and potential applications

**DOI:** 10.1007/s00415-025-12938-z

**Published:** 2025-02-11

**Authors:** Serhat V. Okar, Karan D. Kawatra, Ashley A. Thommana, Daniela C. Vultorius, Govind Nair, María I. Gaitán, Gina Norato, Yair Mina, Anita Fletcher, Daniel S. Reich, Irene Cortese

**Affiliations:** 1 Translational Neuroradiology Section, National Institute of Neurological Disorders and Stroke, National Institute of Health, Bethesda, MD USA; 2 Neuroimmunology Clinic, National Institute of Neurological Disorders and Stroke, National Institutes of Health, Bethesda, MD USA; 3 Experimental Immunotherapeutics Unit, National Institute of Neurological Disorders and Stroke, National Institutes of Health, Building 10, Room 5C103, 10 Center Drive, Bethesda, MD 20814 USA; 4 qMRI Core Facility, National Institute of Neurological Disorders and Stroke, National Institute of Health, Bethesda, MD USA; 5 Office of Biostatistics, National Institute of Neurological Disorders and Stroke, National Institutes of Health, Bethesda, MD USA

**Keywords:** low-field MRI, progressive multifocal leukoencephalopathy, neuroimaging, portable MR

## Abstract

**Background and objective:**

Progressive multifocal leukoencephalopathy (PML) is a severe, disabling infection caused by JC virus reactivation. PML-related disability complicates the MRI monitoring needed to assess treatment interventions in clinical trial or compassionate use settings. Portable ultra-low-field MRI (pULF-MRI) offers a convenient approach when such frequent imaging is needed. We evaluated the potential utility of pULF-MRI as an adjunctive tool for decreasing the burden of clinical study participation and clinical management in PML.

**Methods:**

We examined paired high-field (HF) and pULF-MRI scans from 11 patients, aged 49 ± 15 years. pULF-MRI images with corresponding HF-MRI were coupled to depict key imaging findings of PML, including three patients with longitudinal evaluations, one with bedside pULF-MRI. The images were then independently assessed by two blinded raters, not involved in image acquisition or initial evaluations, who sequentially rated diagnostic accuracy of pULF-MRI scans compared to the HF-MRI. Longitudinal evaluations were performed for three patients, one with bedside pULF-MRI**.**

**Results:**

T2-FLAIR lesions were detected with pULF-ULF in all cases when present on HF-MRI. Median sensitivity and specificity were 62% and 100%, respectively. T1WI hypointense areas showed similar performance. Focal volume loss was present in 8/11 HF-MRI scans, with sensitivity and specificity of detection by pULF-MRI of 100% and 94%, respectively. Contrast enhancement was seen in a single case on both pULF- and HF-MRI. Follow-up pULF-MRI showed lesion changes in two cases, and stable findings in one case, consistent with HF-MRI.

**Discussion:**

pULF-MRI shows promise in evaluation and monitoring of PML, showing moderate-to-high accuracy even when evaluations were unaided by HF-MRI. Our results highlight a potential application of pULF-MRI for facilitating participation in PML clinical research and more generally as a way to reduce burden of clinical management for this disabled patient population.

**Supplementary Information:**

The online version contains supplementary material available at 10.1007/s00415-025-12938-z.

## Introduction

Progressive multifocal leukoencephalopathy (PML) is a debilitating and often life-threatening infection of the central nervous system (CNS) caused by the human polyomavirus JC virus (JCV) [1]. Although it causes a latent, life-long asymptomatic infection in the immune-competent host, suppressed cellular immunity can lead to viral reactivation and PML [1]. HIV-related PML accounts for most cases, with hematological malignancies and treatment with immunomodulatory or immunosuppressive therapies being the next most common associations [1–6].

Magnetic resonance imaging (MRI) is a critical tool for in vivo diagnosis of PML for follow-up [1, 7]. Typical MRI findings of PML include multifocal hyperintense white matter lesions (WML) on T2-weighted fluid-attenuated inversion recovery (T2-FLAIR), mostly involving subcortical and juxtacortical white matter. These lesions are generally isointense and/or hypointense on T1-weighted images (T1WI) depending on the degree of tissue destruction and the type of T1WI [8]. Diffusion-weighted imaging (DWI) hyperintensity at the leading edge of expanding lesions is common [9]. When immune reconstitution is present, MRI can show contrast enhancement, edema, and mass effect, indicating inflammatory changes [10–12]. Asymptomatic PML lesions, detectable through MRI, can develop before clinical symptoms appear or a formal diagnosis is made in natalizumab-treated patients [13, 14]. These lesions often exhibit localized growth, accompanied by edema and contrast enhancement, which may indicate underlying inflammation [15]. JCV CNS infection spectrum can also manifest with granule cell neuronopathy (GCN), causing progressive cerebellar atrophy with or without associated white matter lesions [1, 16, 17], fulminant encephalopathy [18, 19], and meningitis. [1, 20, 21]

New developments in compact, low-cost [22], and portable ultra-low-field (64 millitesla (mT)) MRI (pULF-MRI) scanners have created promising opportunities for point-of-care MRI in both diagnostic and disease monitoring capacities. This technological progress is particularly significant due to challenges associated with accessing HF-MRI scanners related to the considerable neurological disability PML patients commonly experience [3, 23]. Recent studies support the feasibility and diagnostic performance of pULF MRI in intensive care units [24–26] and stroke [27–29] with bedside acquisition. pULF-MRI has also shown to be promising in multiple sclerosis (MS), for detecting WML relative to high-field (HF) MRI (3 T) and show strong correlation in WML burden estimates [30].

As reported in stroke [28, 29] and MS [31, 30], pULF-MRI may help visualize and monitor key disease features in PML, such as confluent WML, contrast enhancement, diffusion changes at lesion edges, and focal or global volume loss.

The elimination of infrastructure requirements and portability of the pULF-MRI scanner [32] positions it as a viable option for the implementation of point-of-care MRI in the context of PML care. Moreover, logistical challenges such as travel burden frequently hinder participation in clinical trials [33, 34], especially pertinent in the case of PML where no FDA-approved treatment is presently available [35]. The adoption of a low-cost, readily accessible MRI can be a way to mitigate barriers, and thereby facilitate participation in clinical trials.

In this study, we assessed the utility of pULF-MRI for PML imaging and demonstrated imaging hallmarks of PML with pULF-MRI. We further examined the sensitivity of pULF-MRI by comparing it to HF-MRI scans acquired on the same day or within a close time frame, with pULF-MRI assessed in a blinded manner (to HF-MRI findings) to better mimic a real-world-like scenario. In addition, we obtained follow-up images using both pULF-MRI and HF-MRI in three cases, including one case with bedside scans, and investigated the evolution of lesions through pULF-MRI in correspondence with HF-MRI findings.

## Materials and method

### Participants

Imaging, demographic, and clinical data were collected with institutional review board approval and after written, informed consent, as part of the National Institute of Neurological Disorders and Stroke’s “Natural History Study of Progressive Multifocal Leukoencephalopathy” protocol (NCT01730131). In this protocol, participants with PML based on 2013 consensus diagnostic criteria [7] undergo standardized longitudinal clinical, radiological, and laboratory monitoring, which includes 3 T MRI of the brain at baseline, and follow-up based on clinical need, with a variety of advanced research pulse sequences.

### MRI acquisition

All MRI acquisitions were performed between July 2021 and July 2023. HF-MRI studies were performed on either of two 3 T Philips Achieva scanners equipped with a 20- or 32-channel head coil, except in two cases (one was scanned on a 3 T Siemens Skyra, other on a 7 T Siemens whole body MRI system). Standard dose gadolinium-based contrast agent (GBCA) (gadobutrol, 0.1 mmol/kg) was administered in all 3 T scans.

pULF-MRI examinations were conducted on protocol participants—either newly enrolled or returning for follow-up—between July 2021 and July 2023. They were given the option to undergo an additional portable 64mT SWOOP MRI system (Hyperfine, Guilford, CT). Imaging details of HF-MRI and pULF-MRI acquisitions are summarized in Table 1.

**Table 1:** Acquisition parameters of pulse sequences in HF-MRI (3 T) and pULF-MRI (64 mT)

Magnetic field strength	Sequence name	Acquisition plane	Repetition time (ms)	Echo time (ms)	Inversion time (ms)	Flip angle (°)	Slice thickness (mm)	Echo train length	Number of slices	Pixel size (mm)	Scan time (min:s)
3 T	T2-FLAIR^a^	Sagittal (3D)	4800	271	1650	90	1.1	182	237	0.7 × 0.7	4:43
3 T	T1WI^a^	Sagittal (3D)	600	28	NA	90	1.1	20	241	0.6 × 0.6	5:15
3 T	MPRAGE	Axial (3D)	7.03	3.2	900	9	0.7	89	241	0.7 × 0.7	7:04
3 T	DWI	Axial	6742	65	NA	90	2.0	55	70	1.7 × 1.7	2:34
3 T	PD-T2WI	Axial	3410	15,100	NA	90	3.0	8	47–47	0.8 × 0.8	3:32
64 mT	T2-FLAIR	Axial (3D)	4000	183	1426	90	5.0	80	36	1.5 × 1.5	11:19
64 mT	T1WI	Axial (3D)	1500	4	300	90	5.0	80	36	1.5 × 1.5	6:24
64 mT	DWI	Axial (3D)	750	93	100	90	6.0	40	30	2.4 × 2.4	16:03
64 mT	T2WI	Axial (3D)	2000	182	NA	90	5.0	24	36	1.6 × 1.6	9:05

*DWI* diffusion-weighted images, *min* minute, *mm* millimeter, *MPRAGE* magnetization-prepared rapid gradient echo, *ms* millisecond, *s* second, *T1WI* T1-weighted images, *T2-FLAIR* T2-weighted fluid-attenuated inversion recovery, *T2WI* T2-weighted images, *PD* proton density

^a^Images acquired before and after gadolinium-based contrast agent administration))

### MRI evaluations

#### Tandem qualitative and quantitative assessments

pULF-MRI and HF-MRI from the closest time point were evaluated in tandem after aligning and spatially co-registering the corresponding sequences from different magnetic field strengths side-by-side via radiology viewing software (Philips Carestream Vue PACS 12.2.2) by a neurologist with 5 years of experience in neuroimaging (SVO). Qualitative assessments were done for each sequence available after slice coupling using the software’s integrated image registration algorithm [36]. HF-MRI slices corresponding to pULF-MRI slices were paired, allowing visualization of consecutive HF-MRI slices between two pULF-MRI slices. This feature enabled SVO to confirm potential lesion correspondence. The main imaging findings (T2-FLAIR lesions, T1WI hypointensity within the PML lesions, contrast enhancement on T1WI, DWI hyperintensity, local volume loss) for both scanners were captured and depicted in the cross-sectional analyses in 11 cases and longitudinal investigations in 3 cases. For WML volume analyses, T2-FLAIR images from pULF-MRI scans were registered to HF-MRI T2-FLAIR images using an automated linear registration tool in ITK-SNAP (Version: 3.8.0, www.itksnap.org/pmwiki/pmwiki.php). Manual adjustments were performed by SVO using anatomical landmarks (anterior commissure, central sulci, Sylvian fissures) to optimize the alignment. The pULF-MRI images were resliced to the slice thickness of HF-MRI images (1 mm). WML masks for each magnetic field strength were manually created by SVO and DCV (supervised by SVO) using the voxel intensity thresholding feature in 3D Slicer (https://www.slicer.org/). WML are categorized into two groups: “Main WML,” consisting of large confluent PML lesions, and “Other WML,” which includes satellite lesions and/or lesions likely unrelated to PML. Each lesion on pULF-MRI was verified by confirming presence of a corresponding area on HF-MRI before segmentation to ensure optimal accuracy.

#### Blinded assessments

In the cross-sectional analysis, two raters (DSR, neuroradiologist with 21 years of experience, and MIG, neurologist with 13 years of experience in neuroimaging) evaluated anonymized pULF-MRI and HF-MRI (via Osirix Dicom Viewer, Version: 13, https://www.osirix-viewer.com/) with a consensus approach in two different sessions with a 2-week washout period between the sessions. Raters reassessed the pULF-MRI scans after a 4-week washout period. For each assessment, they completed an online form detailing findings in 17 anatomical regions (7 supratentorial regions per hemisphere, corpus callosum, cerebellum, and brain stem). The assessment form is provided in Supplemental Fig. 1.

### Statistical analysis

Case-level sensitivity, specificity, positive predictive value (PPV), and negative predictive value (NPV) of pULF-MRI per each individual and for T2-FLAIR hyperintensity, T1WI hypointensity and focal volume loss and were calculated. Intrarater agreement rates were calculated between the initial and second pULF-MRI consensus assessments. Median values and interquartile range (IQR) of case-level sensitivity, specificity, PPV, NPV, and intrarater agreement rates for each imaging feature are reported. Distribution analyses were done using Shapiro–Wilk and Kolmogorov–Smirnov tests. Wilcoxon signed-rank (paired) comparison tests were performed for tandem quantitative WML burden analyses.

## Results

### Cohort characteristics

Eleven adults with PML (mean age ± SD: 49 ± 15 years, female/male: 6/5) participated in the study. Underlying conditions included HIV (participants 1,6,9,10), primary immune deficiency (participant 2), MS treated with natalizumab (participant 8), multiple myeloma (participant 11), ocular pemphigoid treated with rituximab (participant 3), dermatomyositis treated with methotrexate (participant 7), and sarcoidosis (participant 4). Time from initial PML diagnosis to pULF-MRI scan ranged between 1 and 155 months (median 17, IQR 2–84 months). Table 2 summarizes the cohort’s characteristics.

**Table 2:** Demographic and clinical overview of study participants

Participant	Underlying condition	Time between PML diagnosis and pULF-MRI scan (months)	Time between HF-MRI and pULF-MRI scans (days)	Sequences acquired in pULF-MRI
1	HIV	10	0	T2-FLAIR, T1WI, T2WI, DWI
2	Primary immune deficiency	2	0	T2-FLAIR, T1WI, T2WI
3	Ocular pemphigoid treated with rituximab	17	1	T2-FLAIR, T1WI, DWI
4	Sarcoidosis	1	21	T2-FLAIR, T1WI, DWI
5	Primary immune deficiency	89	0	T2-FLAIR, T1WI, DWI
6	HIV	17	0	T2-FLAIR, T1WI, DWI
7	Dermatomyositis treated with methotrexate	24	0	T2-FLAIR, T1WI, T2WI
8	Multiple sclerosis treated with natalizumab	84	0	T2-FLAIR, T1WI
9	HIV	155	2	T2-FLAIR, T1WI, T2WI
10	HIV	5	0	T2-FLAIR, T1WI
11	Multiple myeloma	2	0	T2-FLAIR, T1WI, DWI

*DWI* diffusion-weighted images, *HF-MRI* high-field MRI, *HIV* human immunodeficiency virus, *pULF-MRI* portable ultra-low-field MRI, *T1WI* T1-weighted images, *T2-FLAIR* T2-weighted fluid-attenuated inversion recovery, *T2WI* T2-weighted images

### MRI assessments

Seven participants had pULF-MRI on the same day as HF-MRI. Time between pULF-MRI and HF-MRI scans ranged between 0 and 21 days (median 0 days, IQR 0–2). Two participants had follow-up pULF-MRI, with a total of three scans taken the same day as HF-MRI. All same-day pULF-MRI followed the 3 T HF-MRI scans with GBCA administration done during the HF-MRI scan. Mean GBCA delay (± SD) was 61 (± 24) min for pULF-MRI scans in those following the same-day HF-MRI.

### Cross-sectional analyses of tandem assessments

All participants had T2-FLAIR and T1WI in their pULF-MRI acquisition protocol, whereas DWI was acquired in 6 of 11 patients. T2-weighted imaging (T2WI) was included in 4 of 11 cases, as initial observations suggested no significant additional benefit to justify the longer acquisition time required for T2WI. Mean (± SD) acquisition time for pULF-MRI was 30 (± 7) min. Ten of eleven (90%) cases had T2-FLAIR hyperintense lesions on HF-MRI. In these cases, the primary radiological diagnosis was established by the presence of predominantly confluent T2-FLAIR lesions. In addition, there were smaller scattered lesions observed, which are PML-related satellite lesions [37] and/or unrelated findings. One case presented with isolated cerebellar atrophy without WML, leading to a diagnosis of GCN, and had a corresponding pattern on the pULF-MRI scan (Supplemental Fig. 2).

**Fig. 1:**
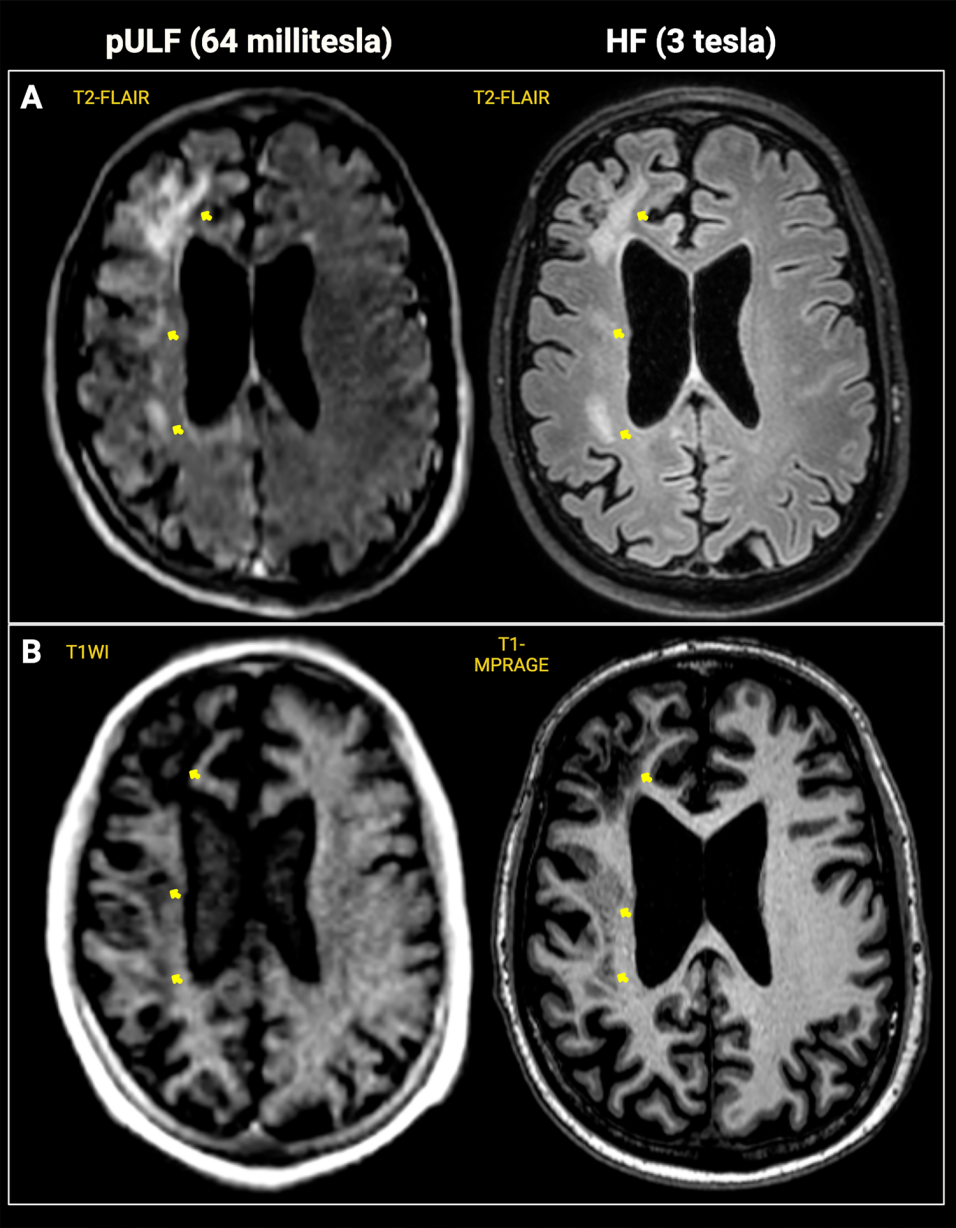
Representative image from a PML case showing pULF-MRI findings that correspond to signal characteristics of the PML lesion at HF-MRI. 61-year-old man with PML and underlying HIV. Extensive PML lesion in the right frontal white matter is visible on both pULF-MRI T2-FLAIR and HF-MRI (3 T) T2-FLAIR images (panel **A**) and both pULF-MRI T1WI and HF-MRI T1-MPRAGE images (panel **B**) with hyperintense and hypointense signal changes, respectively (yellow arrows)

All cases with T2-FLAIR hyperintense lesions on HF-MRI (10/11) exhibited detectable hyperintense lesions on pULF-MRI T2-FLAIR corresponding to the primary confluent T2-FLAIR-hyperintense regions that initially prompted the patients’ diagnosis. 8/11 (72%) patients had T1WI hypointensity within the PML lesions on their HF-MRI images. This was visible on pULF-MRI in seven of eight (87%) cases. Representative images showing T2-FLAIR hyperintense PML lesions with T1WI hypointensity are shown in Fig. 1.

**Fig. 2:**
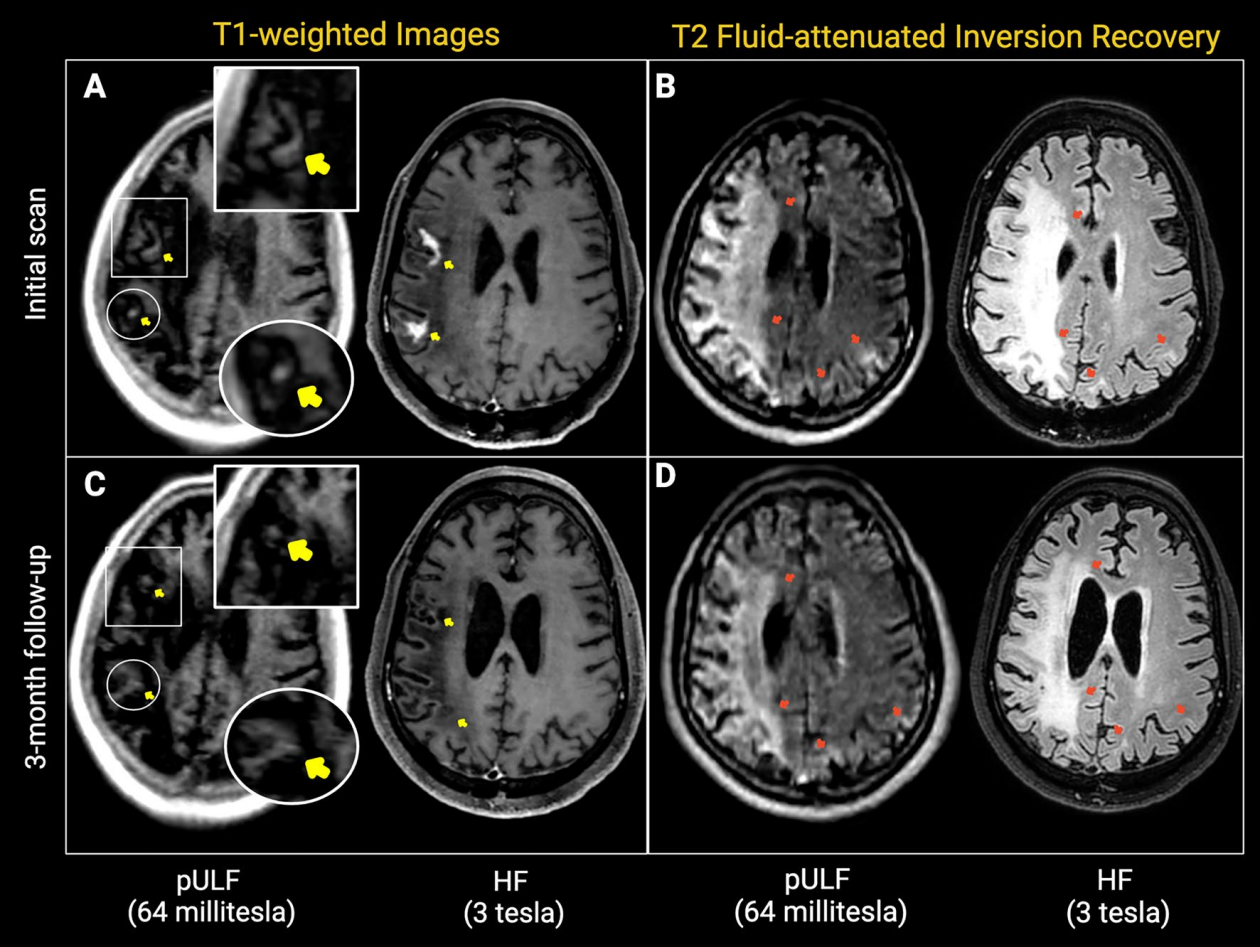
Contrast enhancement and its resolution on follow up scan. 49-year-old man with PML and underlying HIV. Initial (panels **A**, **B**) and 3-month follow up (panels **C**, **D**) pULF-MRI acquired on the same day following HF-MRI are shown. Initial scans showing contrast enhancement on T1W images (yellow arrows; panel **A**) and extensive white matter lesion in the right cerebral hemisphere and left parietal lobe on T2-FLAIR images (red arrows; panel **B**) at both magnetic strength fields. On 3-month follow up, contrast enhancement areas are not visible on T1WI (yellow arrows; panel **C**), and lesion regression with ex vacuo enlargement of the nearby ventricle and sulci on T2-FLAIR (red arrows; panel **D**), are noted at both magnetic fields

Contrast enhancement on T1WI was present in four of ten (40%) cases on HF-MRI scans. Two of these had same-day pULF-MRI, permitting evaluation of post-contrast imaging. Contrast enhancement was not visible in pULF-MRI in one case with punctate contrast enhancement in the PML-satellite lesions, whereas it was identifiable in one case with contrast enhancement within the large confluent PML lesion. Images showing contrast enhancement and resolution of contrast enhancement during the follow-up within the corresponding areas in both magnetic fields are shown in Fig. 2.

DWI hyperintensity (with apparent diffusion coefficient (ADC) isointensity) within the edges of main lesion area was present in 4/10 (40%) cases on 3 T scans. Among the two cases that underwent pULF-MRI on the same day using a protocol including DWI acquisition, DWI hyperintensity pattern was observed in one case across both magnetic fields (Supplemental Fig. 3). Focal volume loss (enlargement of cerebrospinal fluid spaces near the lesion site) was present in 8/11 (72%) cases on HF-MRI and was also visible in the corresponding areas on pULF-MRI.

WML volumes estimated from ten cases exhibiting WML on T2-FLAIR images were not significantly different for pULF-MRI (median, IQR for main WML: 13.6, 7.6–33.5 ml, other WML: 0.15, 0.0–2.9 ml) compared to HF-MRI (median, IQR for main WML: 15.2, 9.0–33.2 ml, other WML: 1.2, 0.2–4.5 ml) (*p* > 0.05 for both categories).

### Cross-sectional analyses of blinded assessments

Table 3 summarizes the diagnostic performance of pULF-MRI for T2-FLAIR lesions, T1WI changes, and local volume loss. Raters detected T2-FLAIR lesions in both pULF-MRI and HF-MRI in all scans exhibiting WML on HF-MRI. Blinded raters also identified the only case without WML correctly without marking T2-FLAIR and T2WI hyperintense lesions and instead marking local volume loss in the infratentorial regions, therefore identifying GCN.

**Table 3:** Summary of the diagnostic performance of portable ultra-low-field (64 millitesla) MRI

Imaging feature	Present in HF-MRI	Present in both pULF-MRI and HF-MRI	Sensitivity, % (median, IQR)	Specificity, % (median, IQR)	PPV, % (median, IQR)	NPV, % (median, IQR)	Intrarater agreement, % (median, IQR)
T2-FLAIR lesions	10/11	10/11	62, 47–89	100, 91–100	100, 80–100	87, 77–100	94, 94–100
T1WI hypointensity	10/11	10/11	67, 50–100	100, 100–100	100, 100–100	91, 77–100	94, 94–100
Local volume loss	8/11	8/11	100, 80–100	94, 92–100	67, 37–100	100, 92–100	94, 88–100

*HF-MRI* high-field MRI, *IQR* interquartile range, *T1WI* T1-weighted images, *T2-FLAIR* T2-weighted fluid-attenuated inversion recovery, *NPV* negative predictive value, *pULF-MRI* portable ultra-low-field MRI, *PPV* positive predictive value

pULF-MRI demonstrated good sensitivity for T2-FLAIR hyperintense and T1WI hypointense lesions, along with high sensitivity for local volume loss. It also exhibited high specificity for these imaging features, with good-to-high PPV and NPV, and high intrarater agreement.

In patients with contrast-enhancing lesions on HF-MRI (4 of 10) who underwent same-day pULF-MRI (2 of 4), blinded raters identified contrast enhancement consistent with the tandem rater. However, blinded raters were unable to detect DWI-ADC patterns (described above in tandem evaluations) in the pULF-MRI scans. Both tandem rater and blinded raters identified lesions in pULF-MRI T2WI images in two patients with T2WI hyperintense lesions visible on HF-MRI.

### Longitudinal follow-up with tandem assessments

**Fig. 3:**
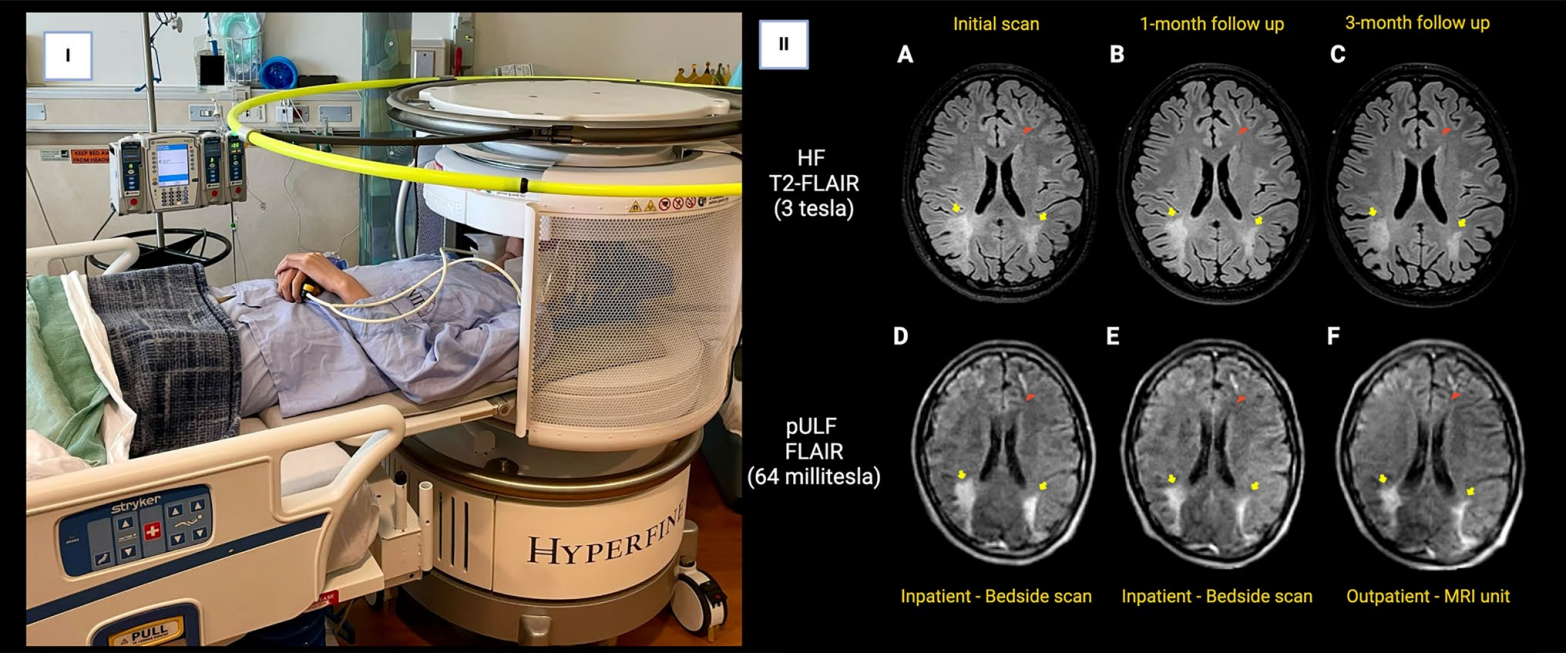
**I** Bedside scan conducted in the inpatient unit following the initiation of immune suppressive treatment. **II** Subsequent MRI follow-up utilizing both magnetic field strengths. The top row (**A**–**C**) displays T2-FLAIR images from HF-MRI (3 T), while the bottom row (**D**–**F**) showcases T2-FLAIR images from pULF-MRI, acquired during the initial scan after IS treatment initiation, and during inpatient/outpatient follow-ups. Minor changes without lesion progression were discernible using pULF-MRI, aligning with the findings from HF-MRI, particularly in the bilateral parieto-occipital regions (arrows). Note that a smaller satellite lesion in the left frontal area was also detectable using pULF-MRI (red arrowheads)

Case 1: A 19-year-old man with primary immune deficiency was diagnosed with PML following suggestive MRI features and the detection of JCV in the CSF. While stabilization of PML was achieved, subsequent conditions required immune suppressive treatment. pULF-MRI was utilized for bedside follow-up for monitoring of possible radiological progression after initiation of immune suppressive treatment. pULF-MRI scans, conducted without GBCA administration, were performed bedside initially and at 1-month follow-up, shifting to outpatient MRI unit visits for 2-month and 3-month follow-ups, all within a 4-week window of the HF-MRI scans. pULF-MRI detected a slight decrease in lesion size over follow-up scans, with no radiological or clinical deterioration despite a modest increase in CSF JCV copy number between the 1-month and 3-month periods. Thus, we successfully monitored the patient using pULF-MRI both at the bedside during post-treatment isolation and during outpatient follow-up visits. Representative images showing a bedside scan in the inpatient room and PML lesions in both magnetic field strengths are shown in Fig. 3.

Case 2: 49-Year-old man with HIV presented with left-sided weakness and seizures. Initial HF-MRI T2-FLAIR showed extensive white matter lesions in the right hemisphere, suggesting PML. A confluent PML lesion had contrast enhancement on post-GBCA T1WI and slight edema, suggesting active inflammation. Same-day pULF-MRI recapitulated these lesions on T2-FLAIR and showed contrast-enhancing areas on T1WI. Follow-up scans in 1 and 3 months showed gradual resolution of the right hemisphere lesion and resolution of contrast enhancement, also visible on the same-day follow up pULF-MRI acquisitions (Fig. 2).

**Fig. 4:**
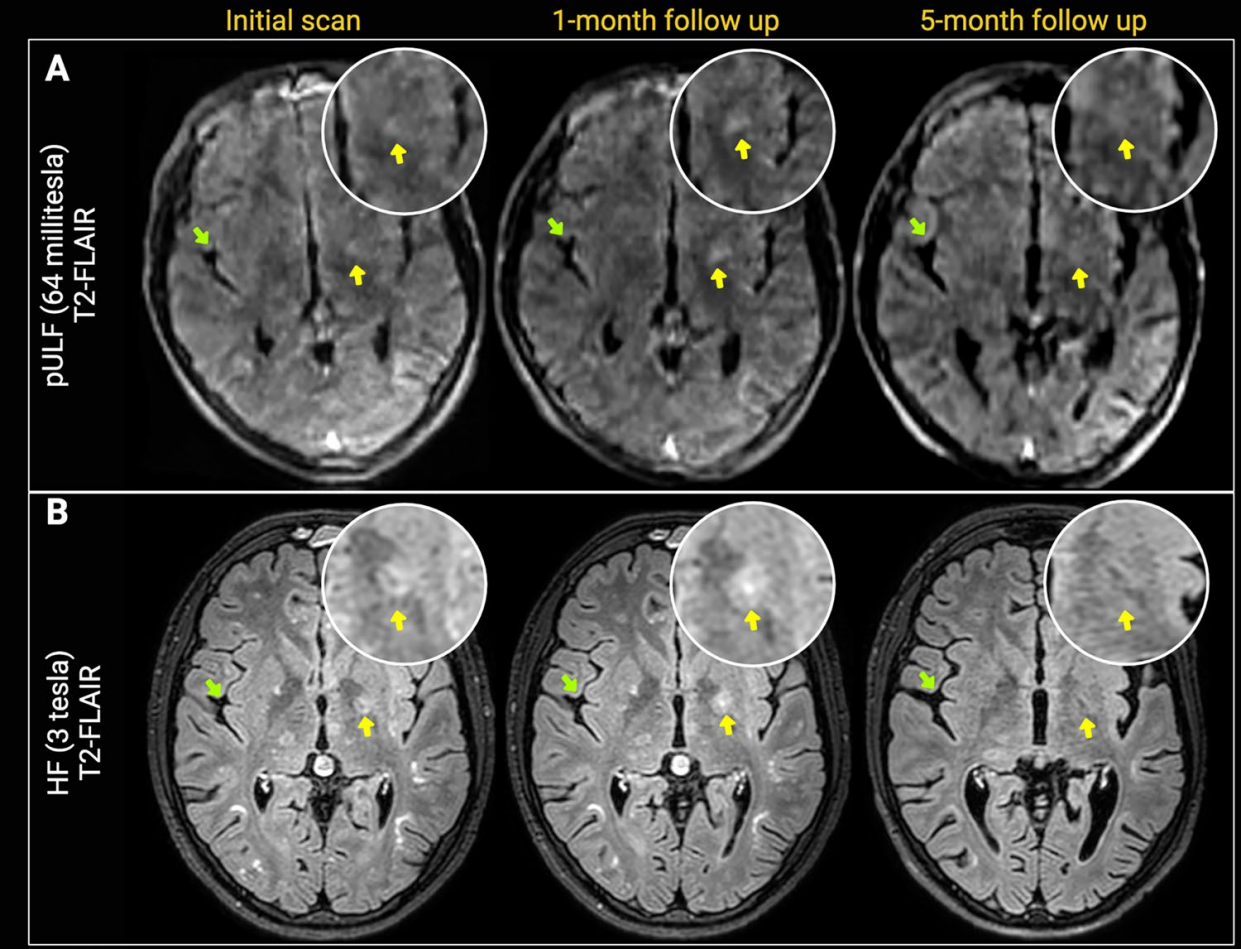
pULF-MRI shows PML lesion progression and regression over time. 21-year-old man with PML and underlying primary immune deficiency. T2-FLAIR images acquired with pULF-MRI (64 miliTesla) (panel **A**) and HF-MRI (3 T) (panel **B**) are shown. pULF-MRI and HF-MRI were acquired within the same week in the initial scan, 1-month and 5-month follow up scans. PML lesion extending to the right deep gray matter area becomes more conspicuous in the 1-month follow up, and regression of this lesion is apparent on 5-month follow-up scans. These changes are visualized in both pULF-MRI and HF-MRI (yellow arrows, magnified view). Enlargement of the CSF spaces and Sylvian fissures (green arrows) suggesting parenchymal volume decrease is visualized on pULF-MRI during the follow-up. Arrows suggesting parenchymal volume decrease is visualized on pULF-MRI during the follow-up

Case 3: A 21-year-old man with primary immune deficiency was diagnosed with asymptomatic PML, based on suggestive MRI and CSF findings. Initial HF-MRI (coupled with pULF-MRI) showed multifocal lesions in bilateral supratentorial and infratentorial areas, most prominently in the left frontal lobe and left temporal lobe, without diffusion restriction. pULF-MRI showed the prominent confluent lesion in the left frontal area as well as additional satellite areas on T2-FLAIR images. Follow-up HF-MRI ~ 1 month after the baseline scan showed lesion progression with extension to the left deep gray matter. Changes on T2-FLAIR images were depicted in the same-day pULF-MRI showing more prominent signal in the left deep gray matter area. Follow-up HF-MRI after 5 months showed lesion regression in the left frontal area with the regression of the left deep gray matter lesion, reflected on the same-day pULF-MRI (both on T2-FLAIR) (Fig. 4).

## Discussion

In this study, we report that pULF-MRI can capture the key imaging characteristics of PML and furthermore, sensitive to imaging findings of PML even if the assessments were performed in an unaided fashion that blinds the raters to HF-MRI findings. On the lesion level, we were able to demonstrate key MRI hallmarks of PML: T2-FLAIR hyperintense lesions containing T1WI hypointense areas associated with local volume loss (tissue damage) and contrast enhancement (active inflammation) [1]. pULF-MRI not only detects PML WML but also provides accurate WML volume estimates when used alongside HF-MRI in tandem assessments. This applies to both large confluent PML lesions and smaller lesions. While the lesion volume detection under HF-MRI-blinded conditions remains unknown, the blinded assessment approach in our study evaluates the accuracy of lesion detection through region-based anatomical analysis. Lesion-level pULF-MRI assessments blinded to HF-MRI findings demonstrated moderate-to-high sensitivity, specificity, PPV, and NPV.

Importantly, we demonstrated the utility and sensitivity of pULF-MRI for longitudinal monitoring of radiological changes in three cases, including one patient with asymptomatic PML and another with bedside follow-up scans. We were also able to demonstrate imaging findings of GCN, which is an atypical type of PML with cerebellar volume loss rather than T2-FLAIR-hyperintense lesions. Finally, we report on a patient exhibiting contrast enhancement, a crucial indicator of active inflammation [8], that subsequently resolved. This highlights the potential of pULF-MRI for frequent radiological monitoring in patients undergoing immune reconstitution, where MRI serves as the most valuable tool for closely monitoring CNS inflammation. [1, 8]

A recent study showed the utility of pULF-MRI in MS [30], some of the disease-modifying treatments for which the risk of PML development is high [38, 39]. Close monitoring of MS patients with high PML risk is crucial. While our study points toward a potential of pULF-MRI as a screening tool in PML, our findings are more applicable to the patients with a confirmed PML diagnosis. However, the good sensitivity we observed, even in blinded settings, suggests that pULF-MRI might be evaluated for diagnostic studies in the future. When baseline pre-PML MRI scans are available, the application of this method could promise reducing the burden of clinical management. Further investigation is needed to assess the reliability of PML screening in pharmacovigilance.

These initial findings emphasize the potential of pULF-MRI for assessing PML, particularly in patients needing frequent monitoring or those facing difficulties accessing traditional MRI due to disability or distance. The introduction of accessible brain imaging technology is critical for improving clinical care and expanding participation in clinical trials, which is especially important given the lack of FDA-approved treatments for PML [35]. Logistical constraints have limited sample sizes in these trials, highlighting the need for portable, low-cost MRI to reduce barriers frequently reported by patients and caregivers [8, 40, 41]. Access to HF-MRI can be constrained due to its high costs [22, 42] and the severe neurological disabilities of PML patients, which can limit their ability to undergo conventional MRI exams. The portability of pULF-MRI offers a solution by enabling bedside imaging, improving access and comfort [43]. Although the small sample size of this initial study limits its generalizability, we were able to capture the spectrum of PML imaging features (T2-FLAIR hyperintensity, contrast enhancement, T1 hypointensity, focal volume loss), highlighting the promising potential of pULF-MRI. These results are consistent with other studies conducted in intensive care [25, 26] and stroke units [27, 28]**,** where patients often face limited access to HF-MRI due to various disease-related challenges. Although pULF-MRI has lower resolution and signal-to-noise ratio, its ability to capture the imaging characteristics of PML may be explained by the fact that PML lesions in our cohort are large and confluent. As the total acquisition time is currently around 30–40 min, there is significant potential to enhance image contrast within acceptable acquisition times, which could improve detection and visualization of smaller lesions that may occur in the earliest stages of the disease. Limitations of the study include the fact that, although we were able to capture same-day acquisitions with both magnetic field strengths in most cases, one case had a considerable time difference between scans, and another case was imaged using a 7 T scanner for the HF-MRI. In addition, the availability of longitudinal data was limited to only three cases. Hence, further validation with larger cohorts and same-day acquisitions is necessary.

In conclusion, pULF-MRI simplifies assessment and monitoring of PML while perhaps also providing a path to easier access to MRI technology in under-resourced settings. These advantages could have important implications by providing enhanced access both to participation in clinical research and patient care in clinical practice.

## Supplementary Information

Below is the link to the electronic supplementary material.

Supplementary file1 (DOCX 11292 KB)

## Data Availability

The authors declare that all data and materials will be available upon reasonable request.

## Abbreviations

CNS: Central nervous system
CSF: Cerebrospinal fluid
GBCA: Gadolinium-based contrast agent
GCN: Granule cell neuronopathy
HF-MRI: High-field MRI
JCV: JC virus
NPV: Negative predictive value
PML: Progressive multifocal leukoencephalopathy
PPV: Positive predictive value
pULF-MRI: Portable ultra-low-field MRI
WML: White matter lesions
