# Supplementary material for: Portable ultra-low-field MRI for progressive multifocal leukoencephalopathy: Case studies, sensitivity, and potential applications
Source: J Neurol. 2025 Feb 11;272(3):193. doi: 10.1007/s00415-025-12938-z (PMC11814002; doi:10.1007/s00415-025-12938-z)
Supplement: Supplementary file 1 — Supplementary file1 (DOCX 11292 KB) [file 415_2025_12938_MOESM1_ESM.docx]

**Appendix**


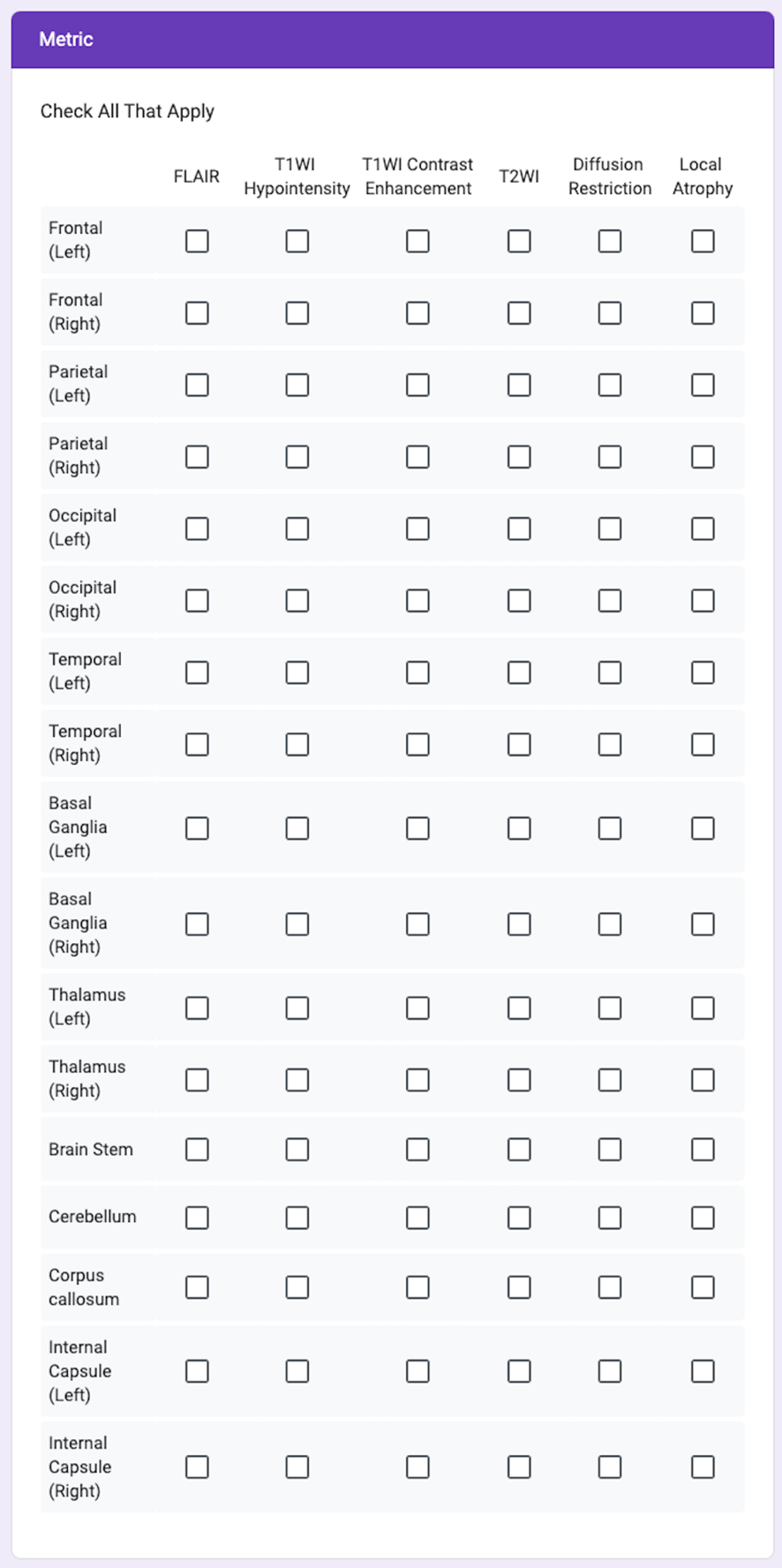


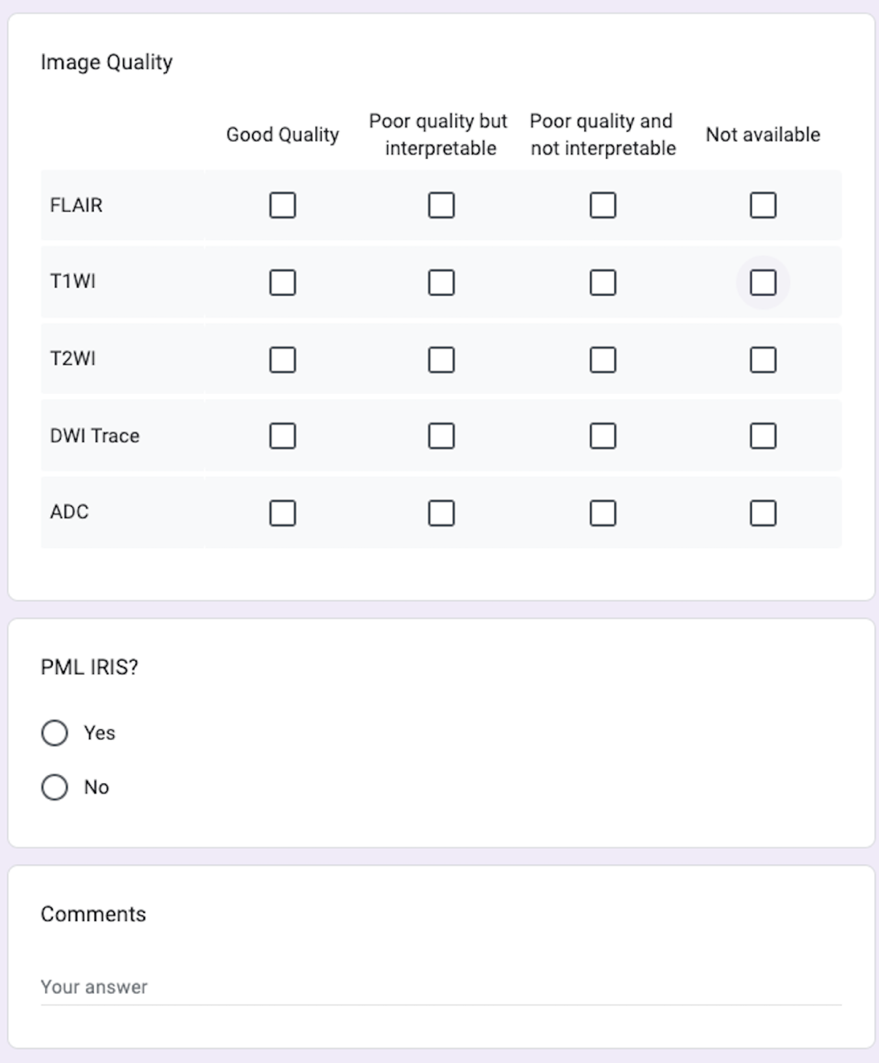


**Supplemental Figure 1:** Online assessment form for the cross-sectional blinded ratings.


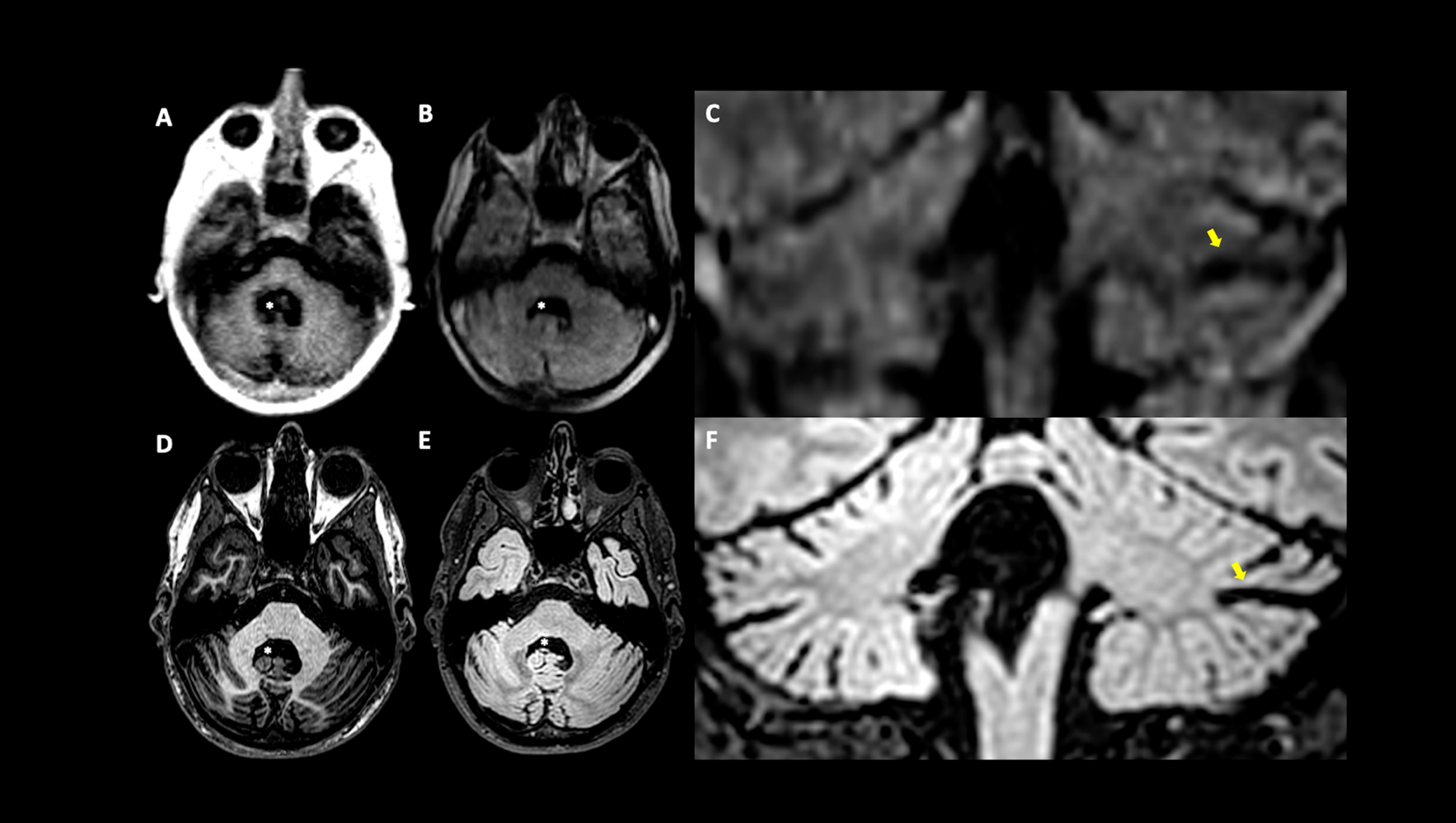


**Supplemental Figure 2:** *Images from a case of JCV granular cell neuronopathy.* 34-year-old woman with progressive cerebellar atrophy and high JCV DNA copy number in the cerebrospinal fluid. Axial T1WI **(A, D)**, axial T2-FLAIR **(B, E),** and coronal T2-FLAIR **(C, F)**. Both pULF-MRI **(A-C)** and HF-MRI (3T) **(D-F)** show enlargement of the fourth ventricle **(white asterisks)** and interlobar fissures **(yellow arrows)** and folia compatible with cerebellar volume loss. (HF-MRI: High-field MRI, T1WI: T1-weighted images, T2-FLAIR: T2-weighted fluid attenuated inversion recovery, pULF-MRI: Portable ultra-low field MRI)


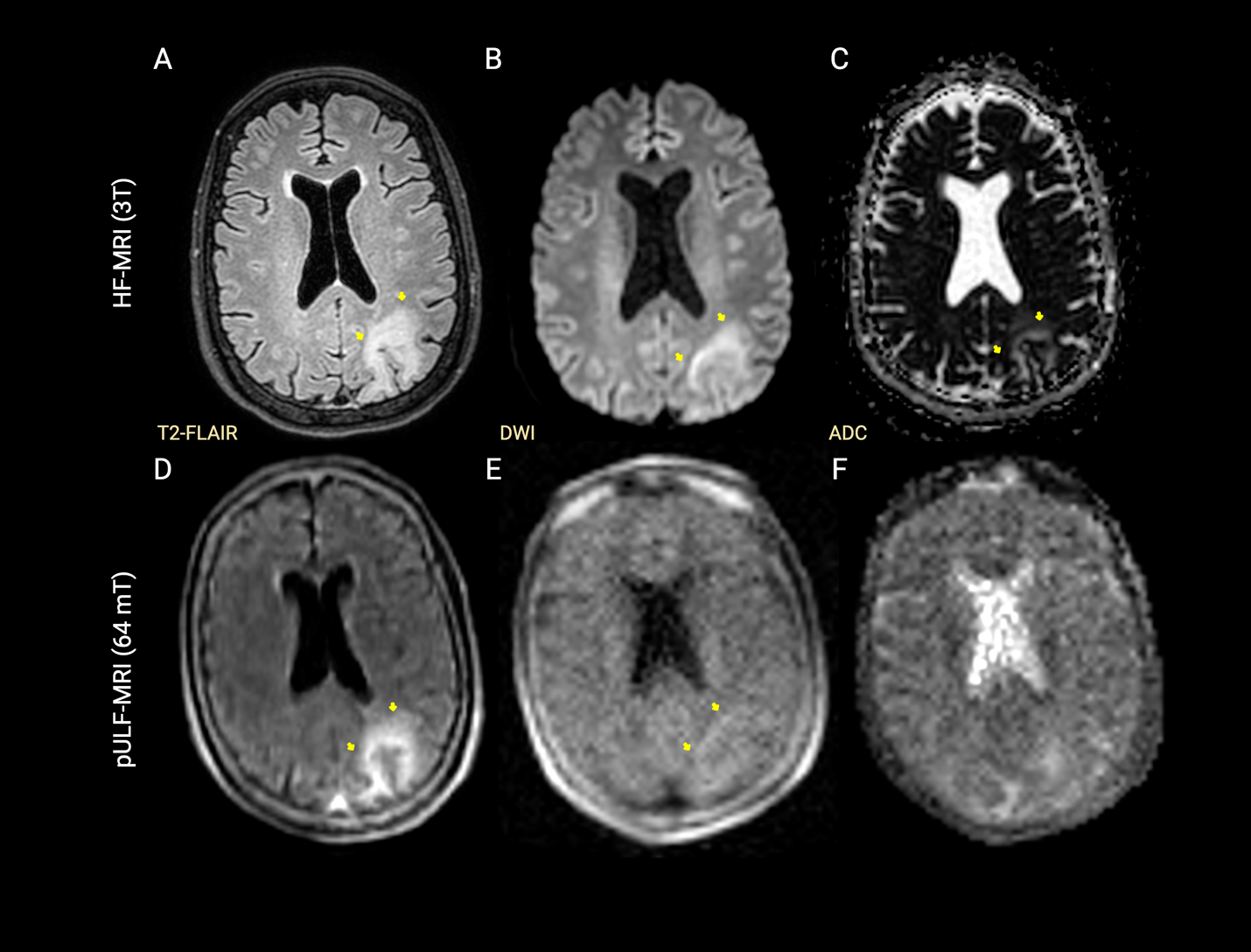


**Supplemental Figure 3:** *DWI findings at the edge of a PML lesion.* A 70-year-old woman with multiple myeloma and PML. Representative images from HF-MRI (A, B, C) and pULF-MRI (D, E, F) acquired on the same day are shown. T2-FLAIR images (A, D) display a large hyperintense PML lesion in the left parietooccipital area (yellow arrows). This lesion exhibits a rim-like hyperintensity on DWI (B) and appears isointense on paired ADC images (C) on HF-MRI (yellow arrows). DWI acquired in pULF-MRI shows hyperintense signal (yellow arrows) without evident ADC changes corresponding to it. (ADC: apparent diffusion coefficient, DWI: Diffusion-weighted imaging, HF-MRI: High-field MRI, pULF-MRI: Portable ultra-low field MRI)
